# Mitotic post-translational modifications of histones promote chromatin compaction *in vitro*

**DOI:** 10.1098/rsob.170076

**Published:** 2017-09-13

**Authors:** Alisa Zhiteneva, Juan Jose Bonfiglio, Alexandr Makarov, Thomas Colby, Paola Vagnarelli, Eric C. Schirmer, Ivan Matic, William C. Earnshaw

**Affiliations:** 1 Wellcome Trust Centre for Cell Biology, School of Biological Sciences, University of Edinburgh, Edinburgh EH9 3BF, UK; 2 Max Planck Institute for Biology of Ageing, Joseph-Stelzmann-Strasse 9b, Cologne 50931, Germany; 3 Institute of Environment, Health and Society, Department of Life Sciences, Brunel University London, Heinz Wolff Building, Uxbridge UB8 3PH, UK

**Keywords:** chromosome compaction, histone modifications, chromatin, mitosis

## Abstract

How eukaryotic chromosomes are compacted during mitosis has been a leading question in cell biology since the nineteenth century. Non-histone proteins such as condensin complexes contribute to chromosome shaping, but appear not to be necessary for mitotic chromatin compaction. Histone modifications are known to affect chromatin structure. As histones undergo major changes in their post-translational modifications during mitotic entry, we speculated that the spectrum of cell-cycle-specific histone modifications might contribute to chromosome compaction during mitosis. To test this hypothesis, we isolated core histones from interphase and mitotic cells and reconstituted chromatin with them. We used mass spectrometry to show that key post-translational modifications remained intact during our isolation procedure. Light, atomic force and transmission electron microscopy analysis showed that chromatin assembled from mitotic histones has a much greater tendency to aggregate than chromatin assembled from interphase histones, even under low magnesium conditions where interphase chromatin remains as separate beads-on-a-string structures. These observations are consistent with the hypothesis that mitotic chromosome formation is a two-stage process with changes in the spectrum of histone post-translational modifications driving mitotic chromatin compaction, while the action of non-histone proteins such as condensin may then shape the condensed chromosomes into their classic mitotic morphology.

## 1. Introduction

Dividing eukaryotic cells must partition their genomes equally between daughter cells. To enable this, interphase chromosomes undergo a dramatic transformation into condensed linear bodies that can be partitioned by the mitotic spindle without becoming tangled or trapped at the division plane. How this transition occurs has been an important question since Flemming's initial description of mitosis [1].

During interphase, chromosome geometry reflects the need to regulate gene expression. Chromosomes occupy distinct territories within the nucleus, their position being correlated with their transcriptional activity. Hi-C experiments showed that each chromosome is divided into ‘compartments’—regions of high or low gene expression [2]. These compartments are further subdivided into topologically associated domains (TADs), which bring distal enhancer sites into close proximity to their cognate promoters. Upon entry into mitosis this hierarchical structure is lost, and the chromosomes instead exhibit global, locus-independent interactions of genome sites less than 10 Mb apart [3].

Several models have been proposed describing the structure of mitotic chromosomes. Early electron microscopy (EM) studies led to a model of chromosomes consisting of chromatin randomly folded like spaghetti [4]. A later model proposed loops organized around a central scaffold of non-histone proteins [5,6]. Alternative models proposed a hierarchical organization of coils or folded domains of increasing size [7,8]. Recent evidence elaborated this theory to suggest that the hierarchically folded domains were organized by a central, condensin rich scaffold [9]. However, *in silico* modelling found that the chromosome organization observed by Hi-C could not be explained by an arrangement of hierarchically folded domains but was probably due to the presence of consecutive, stochastically positioned chromatin loops, of approximately 80 kb [3]. The formation of these loops was not dependent on the presence of a central scaffold; however, such a structure could not be excluded, and could contribute to stabilizing the chromosome axis.

The ‘chromosome scaffold’ was originally identified as a proteinacious structure that could be derived from mitotic chromosomes after nuclease digestion and extraction of most proteins [10]. The known functional components of the chromosome scaffold include condensin [11], topoisomerase II [12] and Kif4A [13]. These proteins are localized along the length of the chromosome axis and contribute to shaping and stabilizing mitotic chromosomes [14]. Condensin is required to re-model *Xenopus* sperm chromatin into mitotic chromatids [15]. However, depletion studies in several species suggest that it is not essential for mitotic chromosome formation *in vivo*, although condensin-depleted chromosomes are structurally compromised, decondensing and forming massive chromatin bridges in anaphase [16–18]. This loss of chromosome integrity can be avoided if protein phosphatase 1 is prevented from targeting to anaphase chromosomes [19]. These results led us to speculate that mitotic chromosome formation might consist of two independent, parallel processes: chromatin compaction and chromosome shaping.

Chromosome shaping involves the disassembly of TADs/compartments and the arrangement of chromatin into loops, possibly through interactions with scaffold components. However, the reasons behind the two- to fourfold compaction that chromatin undergoes during mitotic chromosome formation have not been extensively addressed.

Here, we ask whether histones themselves can drive mitotic chromatin compaction. Early studies showed that the histone N-terminal tails were important for the formation of higher order chromatin structure [20]. Studies using homogeneously modified histones [21] revealed that chromatin compaction can be achieved either through an increase in short-range inter-nucleosomal contacts, e.g. H4 tail interaction with an acidic patch on H2A, or via global chromatin aggregation [21–25].

In this study, we have addressed the mechanisms promoting mitotic chromatin compaction in higher eukaryotes. We reconstituted chromatin using histones isolated from interphase and mitotic cells. These histones contain distinct sets of post-translational modifications (PTMs), as confirmed by mass spectrometry. Microscopy analysis of the assembled chromatin molecules revealed a reproducible increase in the aggregation of chromatin reconstituted with mitotic histones, suggesting that mitotic chromatin compaction could be in part driven by changes in histone PTMs.

## 2. Results

### 2.1. Isolation of histones retaining post-translational modifications

To understand the role of histone PTMs in mitotic chromatin condensation, we adopted an *in vitro* approach, whereby we could detect differences in the compaction of nucleosome arrays reconstituted with histones bearing cell-cycle-regulated PTMs. For this purpose, we purified histones from DT40 chicken lymphoma cells that were either in log phase (≈5% mitosis) or mitotically arrested (80–94% mitosis) and used them to reconstitute chromatin on a tandem array of 25 nucleosome positioning sequences [26].

**Figure 1.**
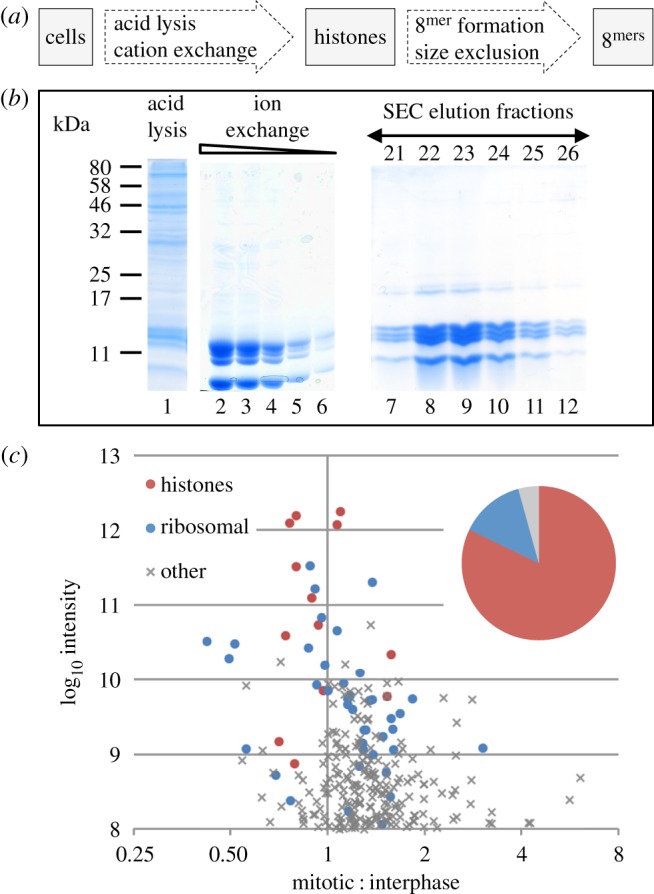
Purification of histones from asynchronous and nocodazole arrested cells. (*a*) A scheme for purification of histones from DT40 cells while preserving cell-cycle-regulated post-translational modifications. (*b*) A typical Coomassie-stained SDS-PAGE gel of purification steps: Lane 1—acid lysis supernatant. Lanes 2–6—dilution series of pooled ion-exchange eluate. Lanes 7–12—elution fractions after size exclusion chromatography (SEC). (*c*) Summary of mass spectrometry (MS) analysis of the isolated histones. Plot of log_10_ of peptide intensities and log_2_ of mitotic enrichment. Red, histones; blue, ribosomal proteins; grey, other contaminating proteins. Inset pie chart shows the total sum of peptide intensities for these proteins.

We isolated histones by acid lysis and ion-exchange chromatography, using a strategy (figure 1*a*) that was previously reported to preserve even labile PTMs such as histone H3 phosphorylation [27,28], which are lost by conventional isolation methods [29]. Figure 1*a,b* shows the isolation strategy and a Coomassie-stained SDS polyacrylamide gel of a typical purification. The initial acid lysis successfully removed many acid insoluble proteins (figure 1*b*, lane 1). Ion-exchange chromatography enriched for core histones, and we removed the majority of linker histones by precipitating the ion-exchange column eluate with perchloric acid. Most of the experiments reported here used histones purified in this way.

### 2.2. Protein composition of histone-containing fractions purified by ion-exchange chromatography

Although highly enriched for histones, low levels of contaminants were present in the protein mixture (figure 1*b*). Mass spectrometry analysis of a SILAC-labelled interphase and mitotic protein mixture identified peptides from 594 protein groups at a false discovery rate (FDR) of 1% and with at least two razor and unique peptides (figure 1*c*). Based on estimated protein abundance from peptide intensities, the histones accounted for 82% of the sample (figure 1*c*). The most abundant contaminating proteins were derived from ribosomes (14% of total peptide intensity). Ribosomal proteins are highly abundant, small and positively charged. The remaining 4% of peptide intensities were derived from 536 other protein contaminants, most of which were equally present in the mitotic and interphase samples. Importantly, the extract did not contain peptides from proteins known to be associated with mitotic chromosome formation (e.g. condensin or cohesin subunits, topoisomerase II or chromokinesins). This allowed us to assay the effects of histone PTMs on chromatin compaction without confounding effects from other factors that are known to play a role in this process.

### 2.3. Characterization of the modification status of the isolated histones

**Figure 2.**
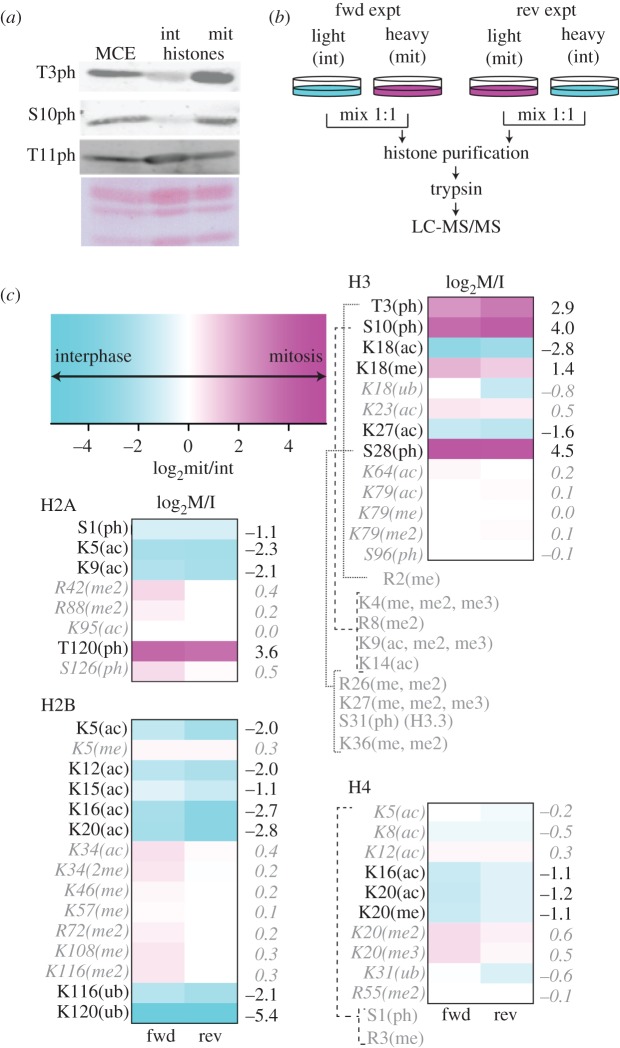
Purified histones preserve their PTMs. (*a*) Immunoblots showing the preservation of labile phosphorylations following acid lysis and ion-exchange chromatography. Ponceau staining of the membrane is shown as a loading control. int, interphase; mit, mitotic. (*b*) A scheme for SILAC MS of histones. Experiments were carried out with forward (fwd) and reverse labelling (rev). (*c*) Summary of cell-cycle-regulated post-PTMs identified by SILAC MS. Colour scale bar for heat-maps showing stage specific enrichment: cyan, interphase enrichment; magenta, mitotic enrichment. Heat-maps show the calculated cell cycle ratio of PTMs on each of the four core histones, from two experiments with forward and reverse labelling (as *b*). Modifications that are enriched more than twofold in either interphase or mitosis are listed in black. Those that remain unchanged are grey and italic. Modifications whose ratio could not be determined as they were seen only on peptides bearing mitotic phosphorylation (e.g. H3R2me) are listed in grey.

To validate this method for purifying histones, we performed immunoblots for several of the most labile PTMs found on mitotic histones. As examples, we focused on three phosphorylation sites of histone H3. H3T3ph is a target of Haspin kinase in mitosis [30]. H3S10ph is phosphorylated by Aurora B kinase in mitosis [31]. H3T11ph is phosphorylated in response to gene activation and is not cell-cycle regulated [32,33]. Figure 2*a* shows that these modifications were readily detected in the isolated histones and that H3T3ph and H3S10ph were substantially enriched in mitosis, whereas H3T11ph was found in both interphase and mitotic histones. Histone H3 phosphorylation is not detected in histones from mitotic chromosomes isolated by conventional methods [29].

To further characterize the global differences in canonical histone PTMs between interphase and mitosis, we performed SILAC experiments (figure 2*b*), using the histone purification protocol described above [27,28] Digested histones were analysed by mass spectrometry and the data analysed by MaxQuant [34]. The identification and quantification information of histone PTMs was obtained from the MaxQuant ‘Sites’ tables, followed by filtering (see Material and methods section).

We display our analysis as heat-maps to highlight changes in PTMs between interphase and mitotic histones (figure 2*c*). Histone H3 phosphorylation of T3, S10 and S28 was highly selective for the mitotic sample, as was phosphorylation of H2A on T120. H4K16ac was selective for interphase histones as previously reported [35]. Also selective for interphase was H2BK120ub, a mark linked to chromatin de-compaction [24,36]. Overall the most dramatic change was observed for histone acetylation, with core histones having from two (H2A) to five (H2B) sites of acetylation that were preferentially enriched in interphase histones.

Marks that did not change between interphase and mitotic histones included H420me2, a mark linked to DNA replication and to CENP-A nucleosomes at centromeres. Also unchanged were H3K27me2/me3, marks characteristic of polycomb chromatin and H3K36me2/me3 found in gene bodies of transcribed genes.

The fact that some histone PTMs coexist in the same peptide complicates the quantification of certain marks. H3R8me2, H3K9me2/me3, H3K9ac and H3K14ac all were apparently increased in mitotic histones. However, these modifications were only detected on peptides that also contained H3S10ph. The unphosphorylated (interphase) peptide was not detected for any of these modifications. As a result, interpretation of the cell cycle distribution of these modifications is ambiguous, although we can conclude that these marks do exist on mitotic histones.

Together, this analysis shows that the dramatic increase in histone phosphorylation that occurs at mitotic entry is accompanied by an equally dramatic programme of histone de-acetylation. Since histone acetylation has long been proposed to be associated with ‘open’ or ‘decompacted’ chromatin, this suggested that mitotic chromatin might have an intrinsic tendency to be more compact than interphase chromatin.

### 2.4. Chromatin reconstitution

**Figure 3.**
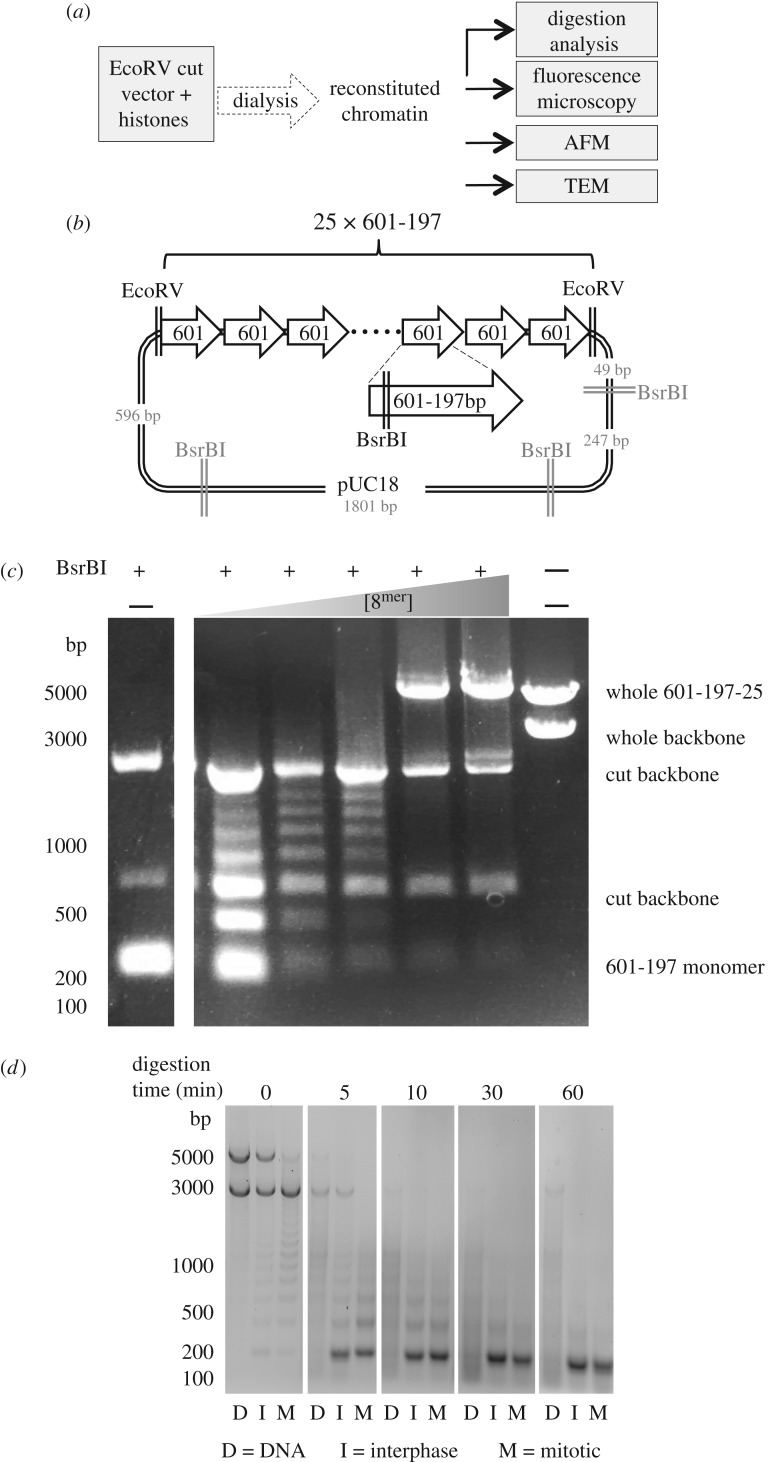
Purified histones can be used to reconstitute chromatin on the 601-197-25 array. (*a*) Scheme of chromatin analysis. After reconstitution the quality of chromatin was assessed by digestion with BsrBI and micrococcal nuclease. Fully assembled chromatin was subjected to analysis by a variety of microscopy methods. (*b*) Vector containing 601-197-25 array. The array is flanked by EcoRV sites. Each 601 monomer contains a BsrBI site. (*c*) BsrBI digestion was carried out on chromatin assembled with an increasing ratio of histones : 601 monomers. Unassembled array was digested into 197 fragments. The presence of a nucleosome on a 601 monomer led to the protection of the BsrBI site, generating longer fragments. When fully saturated the array could no longer be digested. (*d*) Micrococcal nuclease digestion of unreconstituted DNA and chromatin reconstituted with interphase and mitotic derived histones. The presence of an approximately 200 bp ladder confirms the presence of nucleosomes.

Having confirmed the isolation of histones retaining cell-cycle-regulated PTMs, we next prepared uniform populations of chromatin molecules that could be used to test whether those PTMs could promote chromatin aggregation (figure 3*a*). We reconstituted chromatin by dialysing the isolated histones together with a 25^mer^ array of 197 bp Widom-601 sequences (601-197-25) (figure 3*b*), which can assemble highly positioned stable nucleosomes [37]. The array was excised from the vector by digestion, with the undigested plasmid backbone effectively acting as an internal competitor DNA (figure 3*a,b*). Reconstitutions were carried out by overnight dialysis of DNA and histones from 2 M KCl to 0 M KCl buffer.

In order to saturate (but not over-saturate, due to nonspecific assembly of nucleosomes on the DNA) the array with assembled nucleosomes, we set up a titration series of reconstitutions with a variety of histone : DNA ratios. In each case, the extent of reconstitution was monitored by digesting some of the resulting chromatin with a restriction enzyme (BsrBI) that cuts within the nucleosome positioning sequence of naked DNA. When a nucleosome is assembled and positioned on the 601 monomer DNA, it prevents the enzyme from cutting. Thus, progressive assembly of the histone array is detected as a ladder of ascending bands on a DNA gel, ultimately resulting in a band with a mobility of 5000 bp or greater on agarose gels (figure 3*c*). This band corresponds to the fully reconstituted array. This titration experiment was performed for each new histone isolate and all experiments compared arrays that had been reconstituted to a similar extent.

After optimizing the reconstitution conditions, we checked that the chromatin was properly assembled by digestion of both interphase and mitotic chromatin with micrococcal nuclease (figure 3*d*). After a brief digestion, we could see a ladder in the chromatin samples, but not in the naked DNA, which formed a smear. The chromatin was entirely digested to nucleosome core particles after longer treatments with the nuclease. In addition, we digested the array to mono-nucleosomes using AvaI, a restriction enzyme that cuts between monomers in the array, to confirm that the observed decrease in BsrBI digestion efficiency was due to correct chromatin assembly (electronic supplementary material, figure S1). Running the resulting fragments on a native acrylamide gel showed that in the fully saturated arrays that we used for analysis, all the 601 DNA fragments were shifted and no naked, faster running DNA was observed.

We conclude that the reconstitution procedure was successful and complete, with the array assembling an array of phased nucleosomes as originally reported [26].

### 2.5. Analysis of chromatin compaction

**Figure 4.**
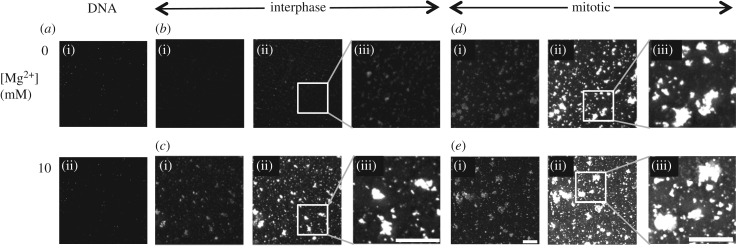
Chromatin reconstituted with histones derived from mitotic cells aggregates even in the absence of divalent cations. (*a*) DNA was absorbed onto poly-l-lysine-coated glass and stained with Hoechst dye. (*b*) Reconstituted interphase chromatin absorbed onto poly-l-lysine-coated glass was stained with Hoechst dye. In all panels, low (i) and high (ii) exposures are shown together with a blow-up of the high exposure (iii). (*c*) Reconstituted interphase chromatin treated with MgCl_2_ was absorbed onto poly-l-lysine-coated glass and stained with Hoechst dye. (*d*) Reconstituted mitotic chromatin was absorbed onto poly-l-lysine-coated glass and stained with Hoechst dye. (*e*) Reconstituted mitotic chromatin treated with MgCl_2_ absorbed onto poly-l-lysine-coated glass and stained with Hoechst dye. Scale bar, 5 µm.

We used fluorescence microscopy to test if chromatin assembled with interphase or mitotic histones exhibited different propensities to aggregate (figure 4). Recently, it was reported that changes in higher order chromatin structure could be assessed by this approach [38]. For this experiment, reconstituted chromatin was adhered onto poly-lysine-coated coverslips and stained with Hoechst dye (figure 4).

When naked DNA fragments were bound to the coverslip, no particles were observed by fluorescence microscopy at any ionic strength (figure 4*a*(i,ii)). This was also the case for chromatin assembled from interphase histones at low ionic strength (figure 4*b*(i)), even at long microscope exposures (figure 4*b*(ii,iii)). By contrast, interphase chromatin exposed to 10 mM MgCl_2_ prior to absorption to the coverslip formed aggregates that were readily observed following staining with Hoechst dye (figure 4*c*(i–iii)). This confirmed previous observations made using a similar method, reconstituting chromatin with chicken erythrocyte histones [38].

A sharp contrast was observed when a similar experiment was conducted using chromatin reconstituted with mitotic histones. Even in the absence of divalent cations, we could clearly see what appeared to be aggregates of chromatin stained with Hoechst dye at high microscope exposures (figure 4*d*(ii,iii)). Similar aggregates were seen with 10 mM magnesium at low and high exposures (figure 4*e*(i–iii)).

These results show that whereas chromatin reconstituted with interphase histones behaved similarly to chromatin reconstituted with chicken erythrocyte histones [38], mitotic histones isolated and reconstituted, in parallel, can promote chromatin assembly into higher order aggregates independent of added divalent cations.

### 2.6. Visualization of mitotic and interphase chromatin by atomic force microscopy

To directly resolve the chromatin aggregates containing mitotic histones, we next examined similar chromatin samples by atomic force microscopy (AFM). After reconstitution, the chromatin was adsorbed onto freshly cleaved mica silanized with (3-aminopropyl)triethoxysilane (APTES) [39]. The samples were then examined using tapping mode microscopy.

**Figure 5.**
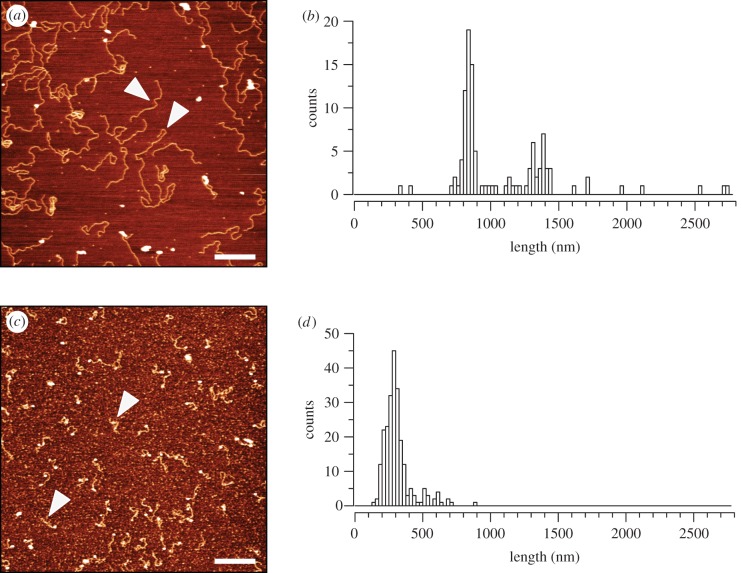
Reconstituted chromatin shows 2.7- to 4.3-fold compaction relative to DNA. Tapping mode AFM was used to image (*a*) DNA or (*c*) interphase chromatin fragments on a silanized mica surface. Presented are representative images. Fragments were measured and the results are presented in the histograms: (*b*) DNA with peaks at approximately 1300 nm (for the 5000 bp 601-197-25 array) and approximately 800 nm (for the 3000 bp vector backbone), *n* = 108 and (*d*) interphase chromatin with peak approximately 300 nm, *n* = 234. White arrowheads point to examples of fragments counted. Scale bar, 500 nm.

To validate our method, initially we observed supercoiled plasmid DNA (electronic supplementary material, figure S2*a*) and EcoRV-digested plasmid containing the 601-197-25 array (figure 5*a*; electronic supplementary material, figure S2*b*). Both samples typically showed mono-disperse populations of DNA particles.

We used ImageJ to measure the length of the fragments that we could see in the digested plasmid sample. Based on the assumption of B form DNA, we expected to observe a mixed population with lengths of approximately 1500 nm (for the 5000 bp 601-197-25 array) and approximately 1000 nm (for the 3000 bp vector backbone). Plotting the lengths of 108 clearly resolved fragments (figure 5*a* arrowheads) revealed two distinct populations: one centred on 1300 nm and the other at around 800 nm, in reasonable agreement with the predicted values (figure 5*b*).

A typical AFM image of chromatin reconstituted with interphase histones is shown in figure 5*c*. The population of interphase chromatin was relatively mono-disperse, but did not show obvious nucleosomes. Although we could not clearly see nucleosome beads on all of the interphase chromatin fragments, when we measured their lengths we saw that the major peak of the distribution was close to 300 nm (figure 5*d*). This is fivefold less than the predicted length of the 601-197-25 array, reflecting the expected degree of compaction if the bulk of the array had been assembled into nucleosomes. We suspect that our failure to resolve individual nucleosomes was due to the fact that the AFM tip used to image these samples was too wide to resolve them.

**Figure 6.**
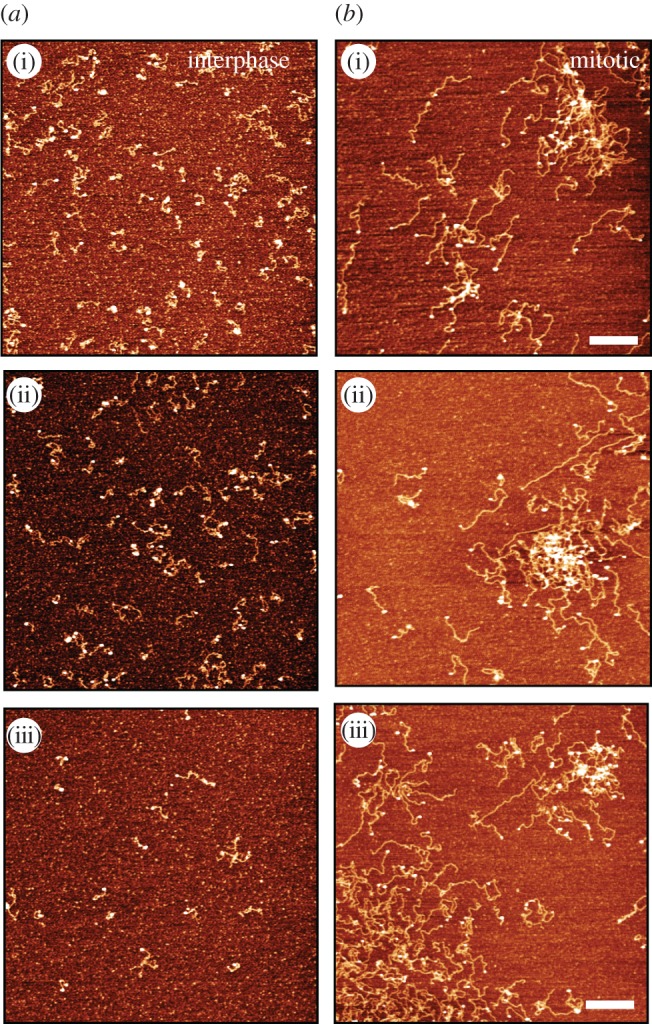
Chromatin fragments assembled with mitotic histones form tangled aggregates. Chromatin reconstituted with interphase (*a*(i–iii)) or mitotic (*b*(i–iii)) histones was adsorbed onto freshly cleaved mica silanized with APTES. Samples were imaged in tapping mode. AFM height analysis of chromatin assembled with interphase histones or mitotic histones. Scale bar, 500 nm.

Chromatin reconstituted with histones from mitotic cells exhibited a dramatically different appearance from that assembled with interphase histones (figure 6). Whereas randomly selected fields of the latter showed predominantly mono-disperse filaments (figure 6*a*(i–iii)), many areas of the mica with mitotic histones had large overlapping aggregates of chromatin (figure 6*b*(i–iii)). Where individual molecules were observed they tended to be collapsed onto themselves. This strongly suggests that the aggregates detected in the mitotic sample by fluorescence microscopy in figure 4 were composed of compacted chromatin. As the only difference between the interphase and mitotic chromatin reconstitutes was in the histones, this suggests that chromatin assembled from mitotic histones exhibits a greater propensity to aggregate under these conditions than chromatin assembled from interphase histones. The most likely explanation for the differences observed between the two chromatin preparations is the different modification status of the histones used.

Although the above results were compelling, we were concerned that non-histone components present in the protein purifications could contribute to the aggregation of chromatin seen in the mitotic samples. In addition, we could not directly observe nucleosomes on the reconstituted chromatin fragments even though micrococcal nuclease digestion and restriction enzyme analysis suggested full reconstitution of the 601-197-25 array.

### 2.7. Visualization of chromatin by transmission electron microscopy

To address these concerns we purified reassembled histone octamers and used transmission electron microscopy (TEM) to visualize the chromatin. NaCl was added to the ion-exchange eluate to form histone octamers that were fractionated by size exclusion chromatography (SEC) [37]. Fractions 21–26 appeared to have an equivalent stoichiometry of each of the four histones and many fewer contaminating proteins (figure 1*b*, lanes 7–12). When analysed by mass spectrometry, we found that after SEC the proportion of peptide intensity derived from histones increased to 98% and 99% for the interphase and mitotic sample, respectively.

Chromatin for TEM analysis was assembled on the 601-197-25 DNA array using histone octamers, mixed with benzylalkyldimethylammonium chloride (BAC) and allowed to adsorb onto carbon-coated grids, with the concentration of chromatin being adjusted based on the A260. The carbon grids were then rotary shadowed with platinum to allow visualization of the chromatin by TEM. As a control for condensed chromatin, we also examined chromatin that had been fixed in the presence of 10 mM MgCl_2_.

**Figure 7.**
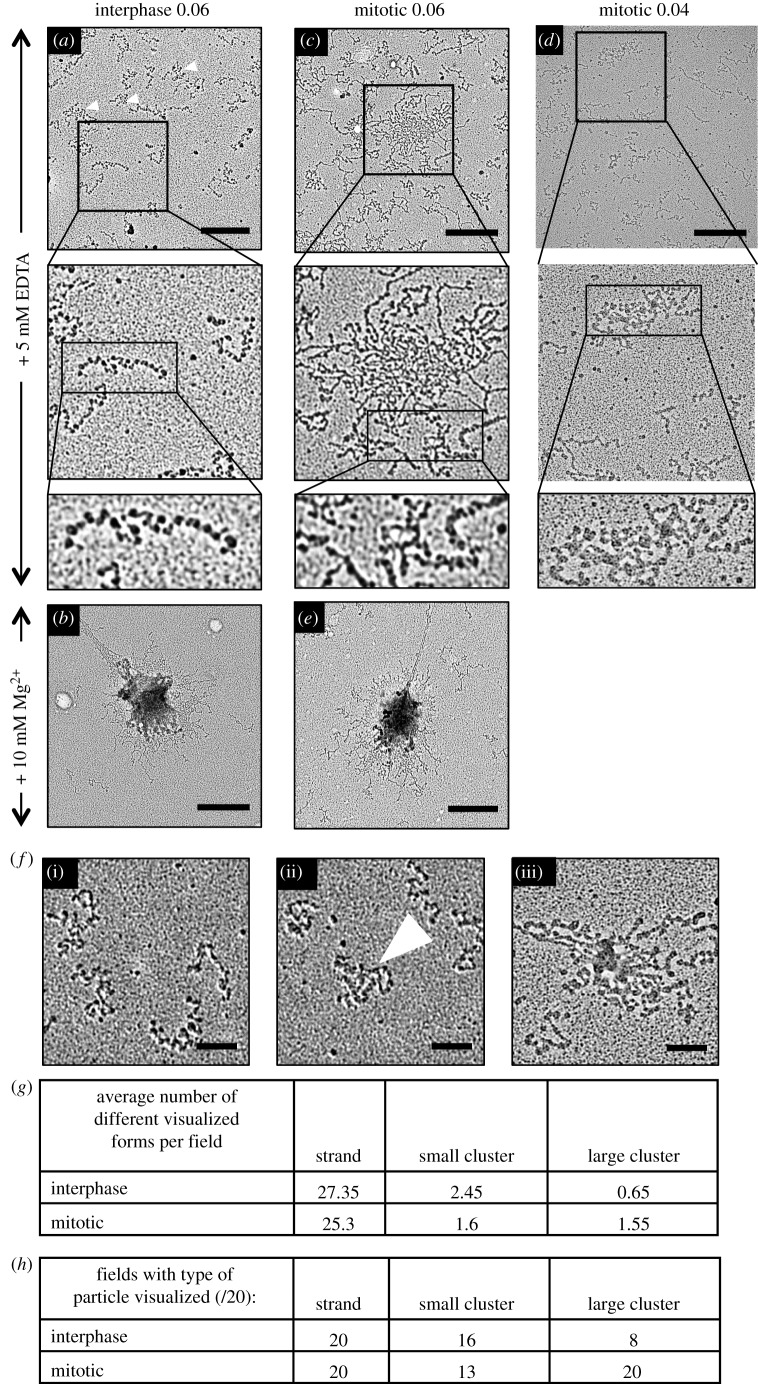
Transmission electron micrographs of reconstituted interphase and mitotic chromatin. Chromatin was mixed with BAC to adhere to carbon-coated grids and rotary shadowed with platinum and imaged by TEM. (*a,b*) Chromatin reconstituted from interphase histones in the absence (*a*) and presence (*b*) of 10 mM Mg^2+^ ions. (*c,d,e*) Chromatin reconstituted from mitotic histones in the absence (*c,d*) and presence (*e*) of 10 mM Mg^2+^ ions. (*a,c,d*) A series of increasing magnifications of a typical field of view. Scale bar, 500 nm. Row 2 = 0.94 µm wide. Row 3 = 0.5 µm wide. (*a–c,e*) Representative images of grids prepared with A260 = 0.06 of chromatin, and (*d*) from a grid prepared with A260 = 0.04 of chromatin. (*f*) Examples of chromatin particles classed as (i) individual strands, (ii) small clusters and (iii) large clusters. Small cluster is indicated with white arrowhead (also three small arrowheads in (*a*)). Scale bar, 100 nm. (*g*) Mean number of particles found per field of view of interphase and mitotic grids. (*h*) Number of fields of view showing respective particle types for interphase and mitotic samples.

In this analysis, the chromatin assembled with interphase histones appeared as mono-disperse ‘beads-on-a-string’ structures (figure 7*a*; electronic supplementary material, figure S3*a,b*). We observed a mixture of DNA fragments with nucleosomes all along their lengths and those that were mostly naked with only a few randomly spaced nucleosomes. We postulate that the former were 601-197-25 arrays saturated with phased nucleosomes, while the latter likely corresponded to plasmid backbone DNA fragments on which nucleosome assembly was less efficient and nucleosome positioning was less ordered. The reconstituted molecules often showed intra-molecular folding (white arrowheads), forming small clusters (see also figure 7*f*(ii)), however we did not see many large aggregates in the interphase sample.

By contrast, interphase chromatin that had been fixed in the presence of 10 mM MgCl_2_ (figure 7*b*—the counterpart of the images shown in figure 4*c*) did form large aggregates, although some individualized chromatin fragments could still be seen (figure 7*b*; electronic supplementary material, figure S3*c,d*). The latter lacked detectable nucleosomes, suggesting that nucleosomes were a driving factor in the formation of aggregates.

When we examined chromatin assembled with isolated mitotic histone octamers, we observed some individual beads-on-a-string fibres similar to those observed in the interphase sample (figure 7*a,c*). However, we also saw many aggregates that resembled those observed with AFM (figure 7*c*; electronic supplementary material, figure S2*c*). As in the case of light microscopy (figure 4*c*,*e*), the MgCl_2_-treated samples of both types of reconstitutes were very similar (figure 7*b,e*). As there was a high density of chromatin seen on the mitotic grids (figure 7*c*), we also present a field from a grid that had less total chromatin adhered to it in order to better resolve aggregated chromatin (figure 7*d*).

Statistical analysis of this experiment confirmed that chromatin assembled with mitotic histones shows a greater propensity to aggregate than does chromatin assembled with interphase histones. To perform this analysis, we lowered the concentration of chromatin adhered to microscope grids, so that the chromatin fragments were more clearly resolved from one another. We examined twenty 4.75 µm^2^ fields for both interphase and mitotic samples in the absence of magnesium ions. We classified visible particles either as individual strands, ‘small clusters’ or ‘large clusters’. Figure 7*f* shows representative chromatin particles from each category. Particles that clearly consisted of many entangled arrays were assigned to the ‘large cluster’ category (figure 7*f*(iii)). Particles assigned to the ‘small cluster’ category were those where a single chromatin fibre could not be clearly resolved either because it showed intra-molecular folding or because the cluster consisted of a small number of fibres compacted together (arrowhead) (figure 7*f*(ii)).

For each condition we classified more than 500 particles (*I* = 609, *M* = 569). We found that although the majority of particles in both conditions appeared as individual strands, there were more than twice as many ‘large clusters’ formed by mitotic chromatin strands as interphase (1.55 versus 0.65 per field) (figure 7*g*). Indeed, whereas all fields in the mitotic sample contained at least one large cluster, most fields in the interphase sample did not (figure 7*h*). When we compared the two samples using the *χ*^2^-test we found a significant difference between the two conditions (*p* = 0.00373).

We conclude that ordered chromatin arrays assembled from mitotic histones exhibit a significantly increased tendency to aggregate into large clusters relative to arrays assembled from interphase chromatin.

## 3. Discussion

We previously reported that mitotic chromosomes can form and segregate in chicken DT-40 cells depleted of at least 95% of their condensin complexes, provided that Repo-Man was prevented from targeting protein phosphatase 1 to anaphase chromatin [19]. At the time, we proposed the existence of an activity termed the regulator of chromosome architecture (RCA) that collaborated with condensin to compact mitotic chromosomes. Subsequent analysis of the protein composition of mitotic chromosomes revealed no obvious candidate for RCA, leading us to hypothesize that it might correspond to the spectrum of post-translational modifications (PTMs) present on core histones during mitosis (see, e.g. the PMM mark [40]). Recent studies of the involvement of Repo-Man in chromatin remodelling further support this hypothesis [41].

Results presented here suggest that changes in the PTMs of core histones may have a significant role in promoting chromatin compaction during mitosis and could correspond to RCA. Although the individual effects of several of the identified modifications on chromatin structure have been investigated previously, their combined effects have not. Because there is such a complex spectrum of histone PTMs, we felt that focusing on the effects of individual modifications could miss the effects of combinatorial changes that might be driving chromatin compaction.

A mass spectrometric survey of PTMs on core histones isolated from both interphase and mitotic cells confirmed the mitotic enrichment of phosphorylations introduced by the Aurora B and Bub 1 kinases (H3S10ph and S28ph). Although many methylations, such as those on H3K27, remained unchanged, we found a dramatic de-acetylation of core histones H2B, H3 and H4 during mitosis. This could potentially trigger higher order chromatin associations by increasing electrostatic interactions mediated by the histone tails.

Recent studies in *Saccharomyces cerevisiae* [42] proposed that mitotic compaction is largely due to increased interactions of the H4 tail with an acidic patch on H2A subsequent to mitotic de-acetylation of H4K16. Introduction of an arginine residue (which cannot be acetylated) at H4 position 16 led to an increase in interphase chromatin compaction. However, despite the finding that loss of Kat8/MOF (the enzyme responsible for H4K16 acetylation) [43] resulted in a global increase of chromatin compaction, developmental changes in H4K16ac did not correlate with changes in chromatin compaction [44]. We suspect that in higher eukaryotes other histone modifications also contribute to mitotic chromatin compaction. Indeed, we confirmed that H4K16 was significantly de-acetylated in mitosis, but several sites on H2B showed an even greater extent of de-acetylation.

Studies *in vitro* have clearly illustrated that a single histone PTM when homogeneously present can dramatically affect the structure of a nucleosome array. *In vivo* we do not expect PTMs to occur at such a high density. However, both Wilkins *et al.* [42] and Li *et al.* [43] demonstrated that changes in physiologically relevant levels of histone modifications could affect chromatin structure.

Based on our finding that mitotic chromatin is compacted in conditions where de-acetylated chromatin assembled with recombinant histones [45] is unfolded (e.g. in the presence of EDTA), we suggest that the combined effect of changing multiple sub-stoichiometric modifications may suffice to promote mitotic chromatin compaction. It is unlikely that the increase in aggregated chromatin is due to the presence of contaminating proteins, because we still saw this phenotype even after SEC fractionation of histones, which reduced non-histone proteins to less than 2% of the protein mixture.

The identity of the PTMs on the histone tails that promote inter-array interactions remains to be determined. Among possible candidates, we observed deubiquitination of H2BK120 in mitosis, which would be expected to promote chromatin compaction [24]. In addition, tri-methylation of H4K20, which has also been linked to chromatin compaction, increased in mitosis, albeit only slightly [23].

Eviction of hyper-phosphorylated linker histone from mitotic chromatin [46] was recently proposed to be a significant factor in driving mitotic compaction [47]. Here, we removed the linker histones from our protein purifications in order to avoid the confounding effects that they exert on higher order structure. Our results suggest that even though the organization of chromatin loops into folded structures (as modelled in Grigoryev *et al.* [47]) is dependent on the presence of linker histone under certain conditions, mitotic chromatin compaction might also be driven, at least in part, by core histone tail interactions.

Our reconstitution protocol aimed to produce uniform chromatin particles where all positioning sequences on the Widom 601 array were occupied by nucleosomes bearing the physiological range of PTMs from interphase and mitotic cells. An unexpected finding was that chromatin reconstituted with mitotic histones formed significantly more aggregates than interphase chromatin, even in the absence of divalent cations. These aggregates, which formed in a low salt buffer containing EDTA, were visualized by several different techniques. This was unprecedented because in previous studies (e.g. [38]) and in our own studies of chromatin reconstituted with interphase histone octamers, aggregation of chromatin fragments was only seen after the addition of at least 4.5 mM Mg^2+^.

The effects of divalent cations on chromatin structure have been investigated since the finding that chromatin purified from rat liver nuclei increased its compaction, from 10 nm to wider solenoidal filaments upon addition of MgCl_2_ [48]. If the linker histones were removed, chromatin did not form organized solenoids, but it still increased in compaction upon addition of magnesium ions. Assembly of chicken erythrocyte nucleosomes on a defined DNA template containing a 12^mer^ array of nucleosome positioning sequences revealed that the chromatin existed in an equilibrium of several soluble folded states [45]. After treatment with magnesium the chromatin was also shown to form insoluble aggregates.

The role of histone N-terminal tails in these structural transitions was examined by using octamers where either H3/H4 or H2A/H2B had been trypsinized [49,50]. Although fully trypsinized octamers failed to oligomerize, and therefore remained soluble in the presence of magnesium ions [49], oligomerization could still occur when H3/H4 tails had been removed by trypsinization [49]. Subsequent studies using recombinant histones (either full length or with the N-terminal tail deleted) highlighted the importance of the H4 N-terminal tail interaction with the acidic patch of histone H2A for intra-molecular chromatin fibre compaction [22].

In our TEM analysis, we identified three classes of particles in both interphase and mitotic samples under conditions where there were no divalent cations present in the buffer: those that were clearly separate, small clusters and large clusters. Particles classed as small clusters could involve several molecules or be the result of intra-molecular folding. Large clusters represent oligomerized/aggregated species. We found that the greatest difference between interphase and mitotic samples was in the frequency of large clusters. As the only differences between the interphase and the mitotic chromatin were in the histone PTMs, we suggest that changes in the PTMs alter the charge properties of the chromatin fibres in such a way as to promote the formation of inter-fibre interactions. *In vivo* this propensity for stabilizing inter-fibre interactions might promote the formation of a polymer-melt structure within mitotic chromatin [51].

We propose that changes in core histone PTMs promote the stabilization of local interactions between chromatin strands. This aggregation, together with the increased fibre flexibility due to decreased linker histone occupancy [47], may promote the formation of chromatin loops that are extruded by condensin complexes to reshape the chromosomes into compact linear bodies. Thus, we propose that mitotic chromosome formation can be divided into two parallel processes: chromatin compaction due to changes in histone post-translational modifications coupled with condensin-dependent shaping.

## 4. Material and methods

### 4.1. Cells

DT40 cells were grown in RPMI medium (Gibco) supplemented with 10% fetal bovine serum (FBS; Labtech), 1% chicken serum (Gibco) and 100 U µl^−1^ penicillin/streptomycin at 39°C, 5% CO_2_. To arrest cells in mitosis, cultures were treated for 12–14 h with 0.5 µg ml^−1^ nocodazole (Sigma-Aldrich). For SILAC labelling cells were grown in RPMI media (Invitrogen) with 10% (v/v) dialysed FBS (Sigma), 100 µg ml^−1^ U-13C615N2-l-lysine : 2HCl and 30 µg ml^−1^ U-13C615N4-l-arginine : HCl (Sigma) for six cell cycles.

To measure the mitotic index, cells were adhered to Polysine slides (VWR). Slides were incubated in 75 mM KCl for 4 min at room temperature and fixed in 75% methanol/25% acetic acid. The slides were inspected under a light microscope and 1000 cells were counted for each condition.

### 4.2. Isolation of histones

Cells (400 × 10^6^) were washed in phosphate buffered saline (PBS) and pelleted. The pellet was resuspended in 0.1 M H_2_SO_4_, incubated at 4°C with rotation for 2 h. The lysate was spun to pellet the cell debris. An equal volume of 1 M Tris–Cl, pH 8 was added to neutralize the supernatant. NaCl to a final concentration 0.5 M, EDTA to a final concentration 2 mM and DTT to a final concentration 1 mM were added to the neutralized lysate.

This neutralized lysate was passed through SP Sepharose fast flow (Sigma) pre-equilibrated with 50 mM Tris–Cl (pH 8.0), 0.5 M NaCl and 2 mM EDTA (pH 8.0). The column was washed with 50 mM Tris–Cl (pH 8.0), 0.6 M NaCl and 2 mM EDTA (pH 8.0). The bound proteins were eluted in 8 × 1.5 ml fractions of 50 mM Tris–Cl (pH 8.0), 2 M NaCl, 2 mM Na-EDTA (pH 8.0). Histones were then precipitated by overnight incubation with 4% perchloric acid at 4°C. They were then washed in 4% perchloric acid acidified acetone and acetone. After this they were resuspended in ddH_2_O plus 0.5 mM PMSF. For SILAC experiments, histones were mixed after purification.

### 4.3. Size exclusion chromatography

Histones were dialysed against refolding buffer (10 mM Tris–Cl pH 7.5, 2 M NaCl, 1 mM EDTA, 5 mM β-mercaptoethanol). The supernatant was concentrated using an Amicon Ultra-0.5 centrifugal filter 10 K NMWL. SEC was performed on a GE Superose 200 increase 300/10 column controlled by an Akta System; 0.5 ml fractions were collected.

### 4.4. SDS-PAGE and immunoblotting

For SDS-PAGE and western blotting 18% SDS were run at 200 V, constant current. SDS polyacrylamide gels were stained with Coomassie Instant Blue (Expedeon). For western blotting, proteins were transferred onto nitrocellulose membrane (GE Healthcare) by wet transfer at 200 mA for 2 h. Membranes were blocked in 5% milk. Primary antibodies were rabbit anti-phospho-histone H3 threonine 3 (H3T3ph) (Millipore) and rabbit anti-phospho-histone H3 serine 10 (H3S10ph) (Cell Signalling Technologies), which were used at 1 : 1000 dilution, and rabbit anti-histone H4 (Abcam), which was used at 1 : 5000 dilution. The secondary antibody was IRDye 800CW goat anti-rabbit (Licor) which was used at 1 : 10 000 dilution. The membranes were imaged using a Licor Odyssey infrared imaging scanner.

### 4.5. Mass spectrometry analysis of histones

#### 4.5.1. Derivatization and enzymatic digestion of histones

SP Sepharose-purified histones were subjected to a propionic anhydride derivatization as previously described [52]. Briefly, histones were resuspended in 1 M ammonium bicarbonate (ABC, pH 8.0) and propionic anhydride 1 : 60 (v/v) propionic anhydride : protein was added. After vortexing and spinning, samples were incubated for 30 min at 51°C. This propionylation step was repeated once. The pH was controlled and adjusted to 8.0 if necessary. After the second propionylation reaction, samples were dried down in a Speedvac concentrator, resuspended in 100 mM ABC and digested in-solution with trypsin 1 : 20 (wt/wt) trypsin : protein ratio overnight at 37°C. Peptides were then desalted using in-house manufactured StageTips [53], dried down in a Speedvac concentrator and resuspended in 0.1% FA before LC-MS/MS analysis.

#### 4.5.2. LC-MS/MS analysis

Liquid chromatography for all LC-MS/MS runs was performed on an EASY-nLC 1000 liquid chromatography system (Thermo Scientific) coupled to a Q Exactive Plus mass spectrometer (Thermo Scientific) via modified NanoFlex sources (Thermo Scientific). Peptides were loaded onto 250-mm × 75-μm PicoFrit (C18 2 µm medium) analytical columns (New Objective) at a maximum pressure of 800 bar. Solutions A and B for the UPLCs were 0.1% formic acid in water and acetonitrile, respectively. Samples were loaded in 0.1% formic acid in water to maximize retention of highly hydrophilic peptides. Gradients varied slightly in length (90–150 min) and mixture, and may be extracted from the respective raw files. In general, they incorporated a linear gradient from very low or zero per cent B to 20 or 30% for 65–100 min, followed by a steeper phase and a wash. This length of gradient was maintained despite the relative simplicity of the protein mixture to improve the resolution and identification of as many modified peptide forms as possible, including those of low abundance. Full-scan MS spectra were acquired from over an *m/z* range of 300–1800 at 70 000 resolution, AGC targets were set to 3 000 000 ions, maximum injection time was 100 ms. MS2 acquisition varied slightly in resolution, AGC target, maximum injection time, and may be extracted from the respective raw files. In general, up to five data-dependent HCD fragmentation MS2 spectra were acquired at a resolution up to 70 000. AGC target for MS2 was set up to 1 000 000 ions. To reach this target, long MS2 injection times were allowed (up to 500 ms). Unassigned, singly-charged or greater than +8-charged ions were rejected and the dynamic exclusion option was enabled (duration: up to 40 s).

#### 4.5.3. Data analysis

Raw files were analysed with MaxQuant proteomics suite of algorithms (v. 1.5.3.17) [34], integrated with the search engine Andromeda [54].

The data were searched against a chicken histone database (generated from a chicken proteome database downloaded on 09.10.2015 from UniProt) with the following parameters: the maximum allowed mass deviation was set to 4.5 ppm for precursor ions and 20 ppm for fragment ions; the minimum peptide length was set to 6 amino acids; the maximum number of missed cleavages was set to 5 with the maximum charge state 6; multiplicity was set to 2 with Lys8/Arg10 as the heavy label and max. labelled AAs were set to 7. Variable modifications included acetylation (Protein N-term and K), methylation (KR), di-methylation (KR), tri-methylation (K), phosphorylation (STY), GlyGly (K)—ubiquitylation remnant on lysine residue after trypsin digestion—and propionylation (K). FTMS top peaks per 100 Da were set to 20.

In-house scripts were used to perform bioinformatic analysis of the MaxQuant processed data. Briefly, for figure 1*c*, the ‘ProteinGroups.txt’ table was loaded and filtered for ‘Potential contaminant’ ≠ ‘+’, ‘Reverse’ ≠ ‘+’, ‘Only identified by site’ ≠ ‘+’ and ‘Razor+unique peptides’ > 2. Intensities and ratios were log_10_ and log_2_ transformed, respectively. For figure 2*c*, ‘Acetyl (K)Sites.txt’, ‘Methyl (KR)Sites.txt’, ‘2Me (KR)Sites.txt’, ‘3Me (K)Sites.txt’, ‘Phospho (STY)Sites.txt’ and ‘GlyGly (K)Sites.txt’ tables were loaded and filtered for ‘Potential contaminant’ ≠ ‘+’, ‘Reverse’ ≠ ‘+’, ‘Localization probability’ > 0.95 and ‘Score’ > 80. Remaining sites were further validated in a manual fashion. Sites not supported by more than two confident spectra (Score > 80 and correct assignment of the modification) were discarded. An additional filtering step was performed. As depicted in figure 2*c*, H3T3ph, H3S10ph and H3S28ph marks are dramatically increased in mitotic cells. Therefore, interpretation of the SILAC ratio of any mark coexisting with the above-mentioned modifications is difficult to interpret. For instance, most of the evidences (and all of them in some cases) for H3R2(me), H3K4(me), H3K4(me2), H3R8(me2), H3K9(ac), H3K9(me), H3K9(me2), H3K9(me3), H3K14(ac), H3R26(me2), H3K27(me), H3K27(me2), H3K27(me3), H3S31(ph) and H3K36(me2) are from peptides that also contain H3T3ph, H3S10ph or H3S28ph and, therefore, were also filtered from the list.

Finally, ratios of filtered marks were log_2_ transformed and plotted as heat-maps, where histone variants were grouped together for clarity, although these are kept separate in the data tables.

### 4.6. Mass spectrometry for size exclusion chromatography purified histone samples

Protein samples were run on a gel and visualized by staining. The stained gel lanes were excised and de-stained and proteins were digested with trypsin, as previously described [55].

Following digestion, samples were diluted with equal volume of 0.1% TFA and spun onto StageTips as described in Rappsilber *et al.* [53]. Peptides were eluted in 40 µl of 80% acetonitrile in 0.1% TFA and concentrated down to 1 µl by vacuum centrifugation. Samples were then prepared for LC-MS/MS analysis by diluting them to 5 µl with 0.1% TFA. MS analysis was performed on an Orbitrap™ Fusion™ Lumos™ tribrid™ mass spectrometer (Thermo Fisher Scientific, UK) coupled online to Ultimate 3000 RSLCnano Systems (Dionex, Thermo Fisher Scientific, UK). Peptides were separated by a PepMap^TM^ RSLC C18 EasySpray column (2 µm, 100 Å, 75 µm × 50 cm) (Thermo Fisher Scientific), operating at 50°C. Mobile phase A consisted of 0.1% formic acid in water while mobile phase B consisted of 80% acetonitrile and 0.1% formic acid. Peptides were loaded onto the column at a flow rate of 0.3 µl min^−1^ and eluted at a flow rate of 0.2 µl min^−1^ according to the following gradient: 2–40% buffer B in 140 min, then to 95% in 11 min. The percentage of buffer B remained at 95 for 5 min and returned back to 2 after one minute. FTMS spectra were recorded at 120 000 resolution (scan range 350–1500 *m/z*) and the peaks with charge greater than or equal to 2 of the MS scan and with an isolation window of 1.4 Thomson were selected and fragmented by higher collisional energy dissociation (HCD) [56] with normalized collision energy of 30. For both MS and MS2 scans, the maximum injection time was set to 50 ms. The AGC targets for MS and MS2 scan were 4.0 × 10^5^ and 2.5 × 10^4^, respectively. The dynamic exclusion was set to 60 s.

The MaxQuant software platform [34] v. 1.5.2.8 was used to process the raw files and search was conducted against the complete *Gallus gallus* proteome (Uniprot, May 2017), using the Andromeda search engine [54]. For the first search, peptide tolerance was set to 20 ppm while for the main search peptide tolerance was set to 4.5 ppm. Isotope mass tolerance was 2 ppm and maximum charge was 7. Digestion mode was set to specific with trypsin allowing maximum of two missed cleavages. Carbamidomethylation of cysteine was set as fixed modification. Oxidation of methionine was set as variable modification. Peptide and protein identifications were filtered to 1% FDR. Absolute protein quantification was determined using the iBAQ algorithm [57].

### 4.7. DNA preparation for chromatin reconstitution

pUC18 containing 25 repeats of the 197-bp Widom 601 sequence (601-197-25) was digested with EcoRV (NEB) to separate the nucleosome binding array from the plasmid backbone.

### 4.8. Chromatin reconstitution

Histone loading onto DNA was achieved by a modified salt gradient method [37]. DNA (0.67 µg µl^−1^) was dialysed together with a titration series of 0.5, 0.8, 1.1, 1.4, 2.0 molar ratio of histone octamers to DNA. Interphase histones had a concentration 0.85 µg µl^−1^, mitotic histones had a concentration 0.732 µg µl^−1^. Chromatin assembly was obtained by overnight dialysis at 4°C in 20 mM HEPES pH 6.5, 5 mM EDTA, 5 mM β-mercaptoethanol, in the presence of a linearly decreasing gradient of KCl, starting at 2 M and ending up at 0 M. Following completion of the gradient, a final 2 h dialysis into fresh low salt buffer was carried out.

### 4.9. Digestion with BsrBI

The degree of reconstitution was monitored by digesting the resulting chromatin with BsrBI (NEB) in CutSmart Buffer at 37°C. The digestion products were visualized by electrophoresis on a 1% agarose gel.

### 4.10. Digestion with AvaI

The degree of reconstitution was confirmed by digesting the resulting chromatin with AvaI (NEB) in CutSmart Buffer at 37°C. The digestion products were visualized by electrophoresis on a 5% acrylamide (37.5 : 1) gel in 0.2× TBE buffer.

### 4.11. Micrococcal nuclease digestion of chromatin

Reconstituted chromatin was fixed with 0.375% formaldehyde for 15 min at room temperature, before addition of CaCl_2_ to a final concentration of 5 mM; 1 U of microccocal nuclease (Worthington) was used for each reaction. Digestion was performed on ice in a buffer containing 50 mM Tris–Cl pH 7.6, 5 mM CaCl_2_, 0.5 mM PMSF, for the indicated time periods, and the reaction was stopped by the addition of excess EGTA. The resulting material was then treated with proteinase K and SDS and incubated at 50°C overnight prior to electrophoresis in a 1% agarose gel and staining with ethidium bromide.

### 4.12. Hoechst dye staining

Reconstituted chromatin was incubated on ice ±10 mM MgCl_2_ for 15 min prior to adsorption to poly-l-lysine-coated coverslips for 15 min. The coverslips were then stained with Hoechst dye and mounted on slides using Vectashield (Vector Labs). The microscope images were acquired on a DeltaVision Core system (Applied Precision) using an inverted Olympus IX-71 stand, with an Olympus UPlan SApo ×100 oil immersion objective (numerical aperture (NA) 1.4) and LED illumination. Camera (Photometrics Cool Snap HQ), shutter and stage were controlled using SoftWorx (Applied Precision). Z-series were collected with a spacing of 0.2 µm, and image stacks were subsequently deconvolved in SoftWorx. Immunofluorescence signals in deconvolved images were analysed using ImageJ software (National Institutes of Health, Bethesda, MD, USA).

### 4.13. Preparation of atomic force microscopy samples

Freshly cleaved mica was silanized by evaporation of (3-aminopropyl)triethoxysilane (APTES; Sigma) in a vacuum chamber [39] for 2 h; 60 µl chromatin samples containing 0.3 µg ml^−1^ DNA were left to adsorb onto the slides for 15 min. The slides were then rinsed with BPC grade H_2_O and dried. An AFM Veeco Nanoman VS with Dimension 3100 controller was used with an NSG10 tip for all measurements (NT-MDT). Images were acquired using tapping mode in air. Images were acquired at 1.19 Hz, 1024 lines/scan. Integral gain: 0.2, proportional gain: 1. Micrographs were generated using Nanoscope v. 3.20 (Veeco) and processed using the Gwyddion v. 2.31 software program to remove horizontal lines and scars.

### 4.14. Transmission electron microscopy

Reconstituted chromatin was fixed with 0.375% formaldehyde at 37°C for 15 min. The reaction was stopped by quenching with Tris–Cl. The A260 of the chromatin was measured and it was diluted in buffer so that the final A260 would be 0.06 or 0.04, and equal for all samples. The lower concentration was used for quantitation experiments because individual particles could be more clearly resolved under these conditions. BAC (Sigma) was added to the chromatin to a concentration of 2 × 10^−4^% (v v^−1^). The mixture was incubated at room temperature for 30 min. The chromatin was spread on formvar/carbon-coated copper grids (TAAB). The grids were washed with ddH_2_O and 90% ethanol and allowed to dry.

For contrast enhancement the grids were rotary shadowed by a Leica ACE600 at a pressure of 1–2.5 × 10^–5^ mbar. Rotating samples were coated with 2 nm platinum (measured by a quartz sensor) at an elevation angle of 7°. The grids were examined by a JEOL JEM-1400 Plus TEM, operated at 80 kV, at 20 000× magnification. Electron micrographs were acquired using a GATAN OneView camera. The resulting images were examined using Digital Micrograph and ImageJ software.

## Supplementary Material

Supplementary Figures

## Data accessibility

The mass spectrometry proteomics data have been deposited to the ProteomeXchange Consortium via the PRIDE partner repository with the dataset identifier PXD007116.

## References

[1] Kschonsak M, Haering CH 2015 Shaping mitotic chromosomes: from classical concepts to molecular mechanisms. BioEssays 37, 755–766. (doi:10.1002/bies.201500020) 25988527 10.1002/bies.201500020 PMC4683672

[2] Lieberman-Aiden E et al. 2009 Comprehensive mapping of long-range interactions reveals folding principles of the human genome. Science 326, 289–293. (doi:10.1126/science.1181369) 19815776 10.1126/science.1181369 PMC2858594

[3] Naumova N, Imakaev M, Fudenberg G, Zhan Y, Lajoie BR, Mirny LA, Dekker J 2013 Organization of the mitotic chromosome. Science 342, 948–953. (doi:10.1126/science.1236083) 24200812 10.1126/science.1236083 PMC4040465

[4] Dupraw EJ 1966 Evidence for a folded-fibre organization in human chromosomes. Nature 209, 577 (doi:10.1038/209577a0) 5921180 10.1038/209577a0

[5] Paulson JR, Laemmli UK 1977 The structure of histone-depleted metaphase chromosomes. Cell 12, 817–828. (doi:10.1016/0092-8674(77)90280-X) 922894 10.1016/0092-8674(77)90280-x

[6] Marsden MPF, Laemmli UK 1979 Metaphase chromosome structure—evidence for a radial loop model. Cell 17, 849–858. (doi:10.1016/0092-8674(79)90325-8) 487432 10.1016/0092-8674(79)90325-8

[7] Sedat J, Manuelidis L 1978 A direct approach to the structure of eukaryotic chromosomes. Cold Spring Harb. Symp. Quant. Biol. 42, 331–350. (doi:10.1101/SQB.1978.042.01.035) 98280 10.1101/sqb.1978.042.01.035

[8] Belmont AS, Sedat JW, Agard DA 1987 A three-dimensional approach to mitotic chromosome structure: evidence for a complex hierarchical organization. J. Cell Biol. 105, 77–92. (doi:10.1083/jcb.105.1.77) 3112167 10.1083/jcb.105.1.77 PMC2114920

[9] Kireeva N, Lakonishok M, Kireev I, Hirano T, Belmont AS 2004 Visualization of early chromosome condensation: a hierarchical folding, axial glue model of chromosome structure. J. Cell Biol. 166, 775–785. (doi:10.1083/jcb.200406049) 15353545 10.1083/jcb.200406049 PMC2172117

[10] Adolph KW, Cheng SM, Paulson JR, Laemmli UK 1977 Isolation of a protein scaffold from mitotic HeLa cell chromosomes. Proc. Natl Acad. Sci. USA 74, 4937–4941. (doi:10.1073/pnas.74.11.4937) 270727 10.1073/pnas.74.11.4937 PMC432072

[11] Saitoh N, Goldberg IG, Wood ER, Earnshaw WC 1994 Scii: an abundant chromosome scaffold protein is a member of a family of putative atpases with an unusual predicted tertiary structure. J. Cell Biol. 127, 303–318. (doi:10.1083/jcb.127.2.303) 7929577 10.1083/jcb.127.2.303 PMC2120196

[12] Earnshaw WC, Halligan B, Cooke CA, Heck M, Liu LF 1985 Topoisomerase-II is a structural component of mitotic chromosome scaffolds. J. Cell Biol. 100, 1706–1715. (doi:10.1083/jcb.100.5.1706) 2985625 10.1083/jcb.100.5.1706 PMC2113886

[13] Mazumdar M, Sundareshan S, Misteli T 2004 Human chromokinesin KIF4A functions in chromosome condensation and segregation. J. Cell Biol. 166, 613–620. (doi:10.1083/jcb.200401142) 15326200 10.1083/jcb.200401142 PMC2172419

[14] Samejima K et al. 2012 Mitotic chromosomes are compacted laterally by KIF4 and condensin and axially by topoisomerase II alpha. J. Cell Biol. 199, 755–770. (doi:10.1083/jcb.201202155) 23166350 10.1083/jcb.201202155 PMC3514791

[15] Shintomi K, Takahashi TS, Hirano T 2015 Reconstitution of mitotic chromatids with a minimum set of purified factors. Nature 17, 1014–1023. (doi:10.1038/ncb3187) 10.1038/ncb3187 26075356

[16] Steffensen S et al. 2001 A role for *Drosophila* SMC4 in the resolution of sister chromatids in mitosis. Curr. Biol 11, 295–307. (doi:10.1016/S0960-9822(01)00096-3) 11267866 10.1016/s0960-9822(01)00096-3

[17] Hagstrom KA, Holmes VF, Cozzarelli NR, Meyer BJ 2002 *C. elegans* condensin promotes mitotic chromosome architecture, centromere organization, and sister chromatid segregation during mitosis and meiosis. Genes Dev. 16, 729–742. (doi:10.1101/gad.968302) 11914278 10.1101/gad.968302 PMC155363

[18] Hudson DF, Vagnarelli P, Gassmann R, Earnshaw WC 2003 Condensin is required for nonhistone protein assembly and structural integrity of vertebrate mitotic chromosomes. Dev. Cell 5, 323–336. (doi:10.1038/nrm1231) 12919682 10.1016/s1534-5807(03)00199-0

[19] Vagnarelli P, Hudson DF, Ribeiro SA, Trinkle-Mulcahy L, Spence JM, Lai F, Farr CJ, Lamond AI, Earnshaw WC 2006 Condensin and Repo-Man-PP1 co-operate in the regulation of chromosome architecture during mitosis. Nat. Cell Biol. 8, 1133–1142. (doi:10.1038/ncb1475) 16998479 10.1038/ncb1475 PMC2741681

[20] Allan J, Harborne N, Rau DC, Gould H 1982 Participation of core histone ‘tails’ in the stabilization of the chromatin solenoid. J. Cell Biol. 93, 285–297. (doi:10.1083/jcb.93.2.285) 7096439 10.1083/jcb.93.2.285 PMC2112843

[21] Shogren-Knaak M, Ishii H, Sun JM, Pazin MJ, Davie JR, Peterson CL 2006 Histone H4-K16 acetylation controls chromatin structure and protein interactions. Science 311, 844–847. (doi:10.1126/science.1124000) 16469925 10.1126/science.1124000

[22] Dorigo B, Schalch T, Bystricky K, Richmond TJ 2003 Chromatin fiber folding: requirement for the histone H4 N-terminal tail. J. Mol. Biol. 327, 85–96. (doi:10.1016/S0022-2836(03)00025-1) 12614610 10.1016/s0022-2836(03)00025-1

[23] Lu X, Simon MD, Chodaparambil JV, Hansen JC, Shokat KM, Luger K 2008 The effect of H3K79 dimethylation and H4K20 trimethylation on nucleosome and chromatin structure. Nat. Struct. Mol. Biol. 15, 1122–1124. (doi:10.1038/nsmb.1489) 18794842 10.1038/nsmb.1489 PMC2648974

[24] Fierz B, Chatterjee C, McGinty RK, Bar-Dagan M, Raleigh DP, Muir TW 2011 Histone H2B ubiquitylation disrupts local and higher-order chromatin compaction. Nat. Chem. Biol. 7, 113–119. (doi:10.1038/nchembio.501) 21196936 10.1038/nchembio.501 PMC3078768

[25] Dhall A, Wei S, Fierz B, Woodcock CL, Lee T.-H, Chatterjee C 2014 Sumoylated human histone H4 prevents chromatin compaction by inhibiting long-range internucleosomal interactions. J. Biol. Chem. 289, 33 827–33 837. (doi:10.1074/jbc.M114.591644) 10.1074/jbc.M114.591644 PMC4256319 25294883

[26] Lowary PT, Widom J 1998 New DNA sequence rules for high affinity binding to histone octamer and sequence-directed nucleosome positioning. J. Mol. Biol. 276, 19–42. (doi:10.1006/jmbi.1997.1494) 9514715 10.1006/jmbi.1997.1494

[27] Rodriguez-Collazo P, Leuba SH, Zlatanova J 2009 Robust methods for purification of histones from cultured mammalian cells with the preservation of their native modifications. Nucleic Acids Res. 37, e81 (doi:10.1093/nar/gkp273) 19443446 10.1093/nar/gkp273 PMC2699528

[28] Leidecker O et al. 2016 Serine is a new target residue for endogenous ADP-ribosylation on histones. Nat. Chem. Biol. 12, 998–1000. (doi:10.1038/nchembio.2180) 27723750 10.1038/nchembio.2180 PMC5113755

[29] Ohta S et al. 2010 The protein composition of mitotic chromosomes determined using multiclassifier combinatorial proteomics. Cell 142, 810–821. (doi:10.1016/j.cell.2010.07.047) 20813266 10.1016/j.cell.2010.07.047 PMC2982257

[30] Dai J 2005 The kinase haspin is required for mitotic histone H3 Thr 3 phosphorylation and normal metaphase chromosome alignment. Genes Dev. 19, 472–488. (doi:10.1101/gad.1267105) 15681610 10.1101/gad.1267105 PMC548948

[31] Hsu JY et al. 2000 Mitotic phosphorylation of histone H3 is governed by Ipl1/aurora kinase and Glc7/PP1 phosphatase in budding yeast and nematodes. Cell 102, 279–291. (doi:10.1016/S0092-8674(00)00034-9) 10975519 10.1016/s0092-8674(00)00034-9

[32] Metzger E et al. 2008 Phosphorylation of histone H3 at threonine 11 establishes a novel chromatin mark for transcriptional regulation. Nature 10, 53–60. (doi:10.1038/ncb1668) 10.1038/ncb1668 PMC2878724 18066052

[33] Yang W et al. 2012 PKM2 phosphorylates histone H3 and promotes gene transcription and tumorigenesis. Cell 150, 685–696. (doi:10.1016/j.cell.2012.07.018) 22901803 10.1016/j.cell.2012.07.018 PMC3431020

[34] Cox J, Mann M 2008 MaxQuant enables high peptide identification rates, individualized p.p.b.-range mass accuracies and proteome-wide protein quantification. Nat. Biotechnol. 26, 1367–1372. (doi:10.1038/nbt.1511) 19029910 10.1038/nbt.1511

[35] Kruhlak MJ, Hendzel MJ, Fischle W, Bertos NR, Hameed S, Yang XJ, Verdin E, Bazett-Jones DP 2001 Regulation of global acetylation in mitosis through loss of histone acetyltransferases and deacetylases from chromatin. J. Biol. Chem. 276, 38 307–38 319. (doi:10.1074/jbc.M100290200) 10.1074/jbc.M100290200 11479283

[36] Debelouchina GT, Gerecht K, Muir TW 2017 Ubiquitin utilizes an acidic surface patch to alter chromatin structure. Nat. Chem. Biol. 13, 105–110. (doi:10.1038/nchembio.2235) 27870837 10.1038/nchembio.2235 PMC5161692

[37] Rogge RA, Kalashnikova AA, Muthurajan UM, Porter-Goff ME, Luger K, Hansen JC 2013 Assembly of nucleosomal arrays from recombinant core histones and nucleosome positioning DNA. J. Vis. Exp. 10, e50354 (doi:10.3791/50354) 10.3791/50354 PMC3864267 24056546

[38] Maeshima K et al. 2016 Nucleosomal arrays self-assemble into supramolecular globular structures lacking 30-nm fibers. EMBO J. 35, 1115–1132. (doi:10.15252/embj.201592660) 27072995 10.15252/embj.201592660 PMC4868957

[39] Wang H, Bash R, Yodh JG, Hager GL, Lohr D, Lindsay SM 2002 Glutaraldehyde modified mica: a new surface for atomic force microscopy of chromatin. Biophys. J. 83, 3619–3625. (doi:10.1016/S0006-3495(02)75362-9) 12496129 10.1016/S0006-3495(02)75362-9 PMC1302437

[40] Markaki Y, Christogianni A, Politou AS, Georgatos SD 2009 Phosphorylation of histone H3 at Thr3 is part of a combinatorial pattern that marks and configures mitotic chromatin. J. Cell Sci. 122, 2809–2819. (doi:10.1242/jcs.043810) 19622635 10.1242/jcs.043810

[41] de Castro IJ et al. 2017 Repo-Man/PP1 regulates heterochromatin formation in interphase. Nat. Commun. 8, 14048 (doi:10.1038/ncomms14048) 28091603 10.1038/ncomms14048 PMC5241828

[42] Wilkins BJ, Rall NA, Ostwal Y, Kruitwagen T, Hiragami-Hamada K, Winkler M, Barral Y, Fischle W, Neumann H 2014 A cascade of histone modifications induces chromatin condensation in mitosis. Science 343, 77–80. (doi:10.1126/science.1244508) 24385627 10.1126/science.1244508

[43] Li X, Li L, Pandey R, Byun JS, Gardner K, Qin Z, Dou Y 2012 The histone acetyltransferase MOF is a key regulator of the embryonic stem cell core transcriptional network. Cell Stem Cell 11, 163–178. (doi:10.1016/j.stem.2012.04.023) 22862943 10.1016/j.stem.2012.04.023 PMC3413170

[44] Taylor GCA, Eskeland R, Hekimoglu-Balkan B, Pradeepa MM, Bickmore WA 2013 H4K16 acetylation marks active genes and enhancers of embryonic stem cells, but does not alter chromatin compaction. Genome Res. 23, 2053–2065. (doi:10.1101/gr.155028.113) 23990607 10.1101/gr.155028.113 PMC3847775

[45] Schwarz PM, Hansen JC 1994 Formation and stability of higher-order chromatin structures: contributions of the histone octamer. J. Biol. Chem. 269, 16 284–16 289. 8206934

[46] Bleher R, Martin R 1999 Nucleo-cytoplasmic translocation of histone H1 during the HeLa cell cycle. Chromosoma 108, 308–316. (doi:10.1007/s004120050382) 10525967 10.1007/s004120050382

[47] Grigoryev SA, Bascom G, Buckwalter JM, Schubert MB, Woodcock CL, Schlick T 2016 Hierarchical looping of zigzag nucleosome chains in metaphase chromosomes. Proc. Natl Acad. Sci. USA 113, 1238–1243. (doi:10.1073/pnas.1518280113) 26787893 10.1073/pnas.1518280113 PMC4747710

[48] Finch JT, Klug A 1976 Solenoidal model for superstructure in chromatin. Proc. Natl Acad. Sci. USA 73, 1897–1901. (doi:10.1073/pnas.73.6.1897) 1064861 10.1073/pnas.73.6.1897 PMC430414

[49] Schwarz PM, Felthauser A, Fletcher TM, Hansen JC 1996 Reversible oligonucleosome self-association: dependence on divalent cations and core histone tail domains. Biochemistry 35, 4009–4015. (doi:10.1021/bi9525684) 8672434 10.1021/bi9525684

[50] Tse C, Hansen JC 1997 Hybrid trypsinized nucleosomal arrays: identification of multiple functional roles of the H2A/H2B and H3/H4 N-termini in chromatin fiber compaction. Biochemistry 36, 11 381–11 388. (doi:10.1021/bi970801n) 10.1021/bi970801n 9298957

[51] Eltsov M, MacLellan KM, Maeshima K, Frangakis AS, Dubochet J 2008 Analysis of cryo-electron microscopy images does not support the existence of 30-nm chromatin fibers in mitotic chromosomes in situ. Proc. Natl Acad. Sci. USA 105, 19 732–19 737. (doi:10.1073/pnas.0810057105) 10.1073/pnas.0810057105 PMC2604964 19064912

[52] Garcia BA, Mollah S, Ueberheide BM, Busby SA, Muratore TL, Shabanowitz J, Hunt DF 2007 Chemical derivatization of histones for facilitated analysis by mass spectrometry. Nat. Protoc. 2, 933–938. (doi:10.1038/nprot.2007.106) 17446892 10.1038/nprot.2007.106 PMC4627699

[53] Rappsilber J, Ishihama Y, Mann M 2003 Stop and go extraction tips for matrix-assisted laser desorption/ionization, nanoelectrospray, and LC/MS sample pretreatment in proteomics. Anal. Chem. 75, 663–670. (doi:10.1021/ac026117i) 12585499 10.1021/ac026117i

[54] Cox J, Neuhauser N, Michalski A, Scheltema RA, Olsen JV, Mann M 2011 Andromeda: a peptide search engine integrated into the MaxQuant environment. J. Proteome Res. 10, 1794–1805. (doi:10.1021/pr101065j) 21254760 10.1021/pr101065j

[55] Shevchenko A, Wilm M, Vorm O, Mann M 1996 Mass spectrometric sequencing of proteins from silver-stained polyacrylamide gels. Anal. Chem. 68, 850–858. (doi:10.1021/ac950914h) 8779443 10.1021/ac950914h

[56] Olsen JV, Macek B, Lange O, Makarov A, Horning S, Mann M 2007 Higher-energy C-trap dissociation for peptide modification analysis. Nat. Meth. 4, 709–712. (doi:10.1038/nmeth1060) 10.1038/nmeth1060 17721543

[57] Schwanhäusser B, Busse D, Li N, Dittmar G, Schuchhardt J, Wolf J, Chen W, Selbach M 2011 Global quantification of mammalian gene expression control. Nature 473, 337–342. (doi:10.1038/nature10098) 21593866 10.1038/nature10098

