# Supplementary material for: Mitotic post-translational modifications of histones promote chromatin compaction in vitro
Source: Open Biol. 2017 Sep 13;7(9):170076. doi: 10.1098/rsob.170076 (PMC5627050; doi:10.1098/rsob.170076)
Supplement: Supplementary Figures [file rsob170076supp1.pdf]

Supplementary Figures

"**Mitotic post-translational modifications of histones promote chromatin compaction in vitro**"

Alisa Zhiteneva, Juan Jose Bonfiglio, Alexandr Makarov, Thomas Colby, Paola Vagnarelli, Eric C. Schirmer, Ivan Matic and William C. Earnshaw

doi: 10.1098/rsob.20170076

Abstract

How eukaryotic chromosomes are compacted during mitosis has been a leading question in cell biology since the nineteenth century. Non-histone proteins such as condensin complexes contribute to chromosome shaping, but appear not to be necessary for mitotic chromatin compaction. Histone modifications are known to affect chromatin structure. As histones undergo major changes in their post-translational modifications during mitotic entry, we speculated that the spectrum of cell cycle-specific histone modifications might contribute to chromosome compaction during mitosis. To test this hypothesis, we isolated core histones from interphase and mitotic cells and reconstituted chromatin with them. We used mass spectrometry to show that key post-translational modifications remained intact during our isolation procedure. Light, atomic force and transmission electron microscopy analysis showed that chromatin assembled from mitotic histones has a much greater tendency to aggregate than chromatin assembled from interphase histones even under low magnesium conditions where interphase chromatin remains as separate beads-on-a-string structures. These observations are consistent with the hypothesis that mitotic chromosome formation is a two-stage process with changes in the spectrum of histone posttranslational modifications driving mitotic chromatin compaction, while the action of non-histone proteins such as condensin may then shape the condensed chromosomes into their classic mitotic morphology

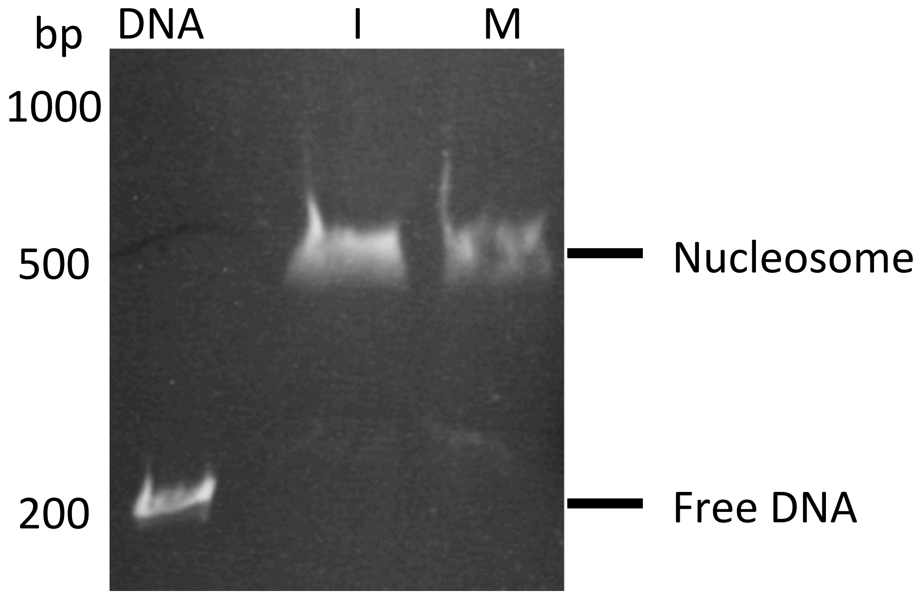

Figure S1. DNA and reconstituted chromatin digested in the linker region. Native PAGE of fragments of 601-197-25 array, reconstituted with interphase (I) or mitotic (M) histones and digested with a restriction endonuclease that cuts within the linker region between nucleosome positioning sequences. Correctly assembled and chromatin that is phased on the array is released as mononucleosomes.

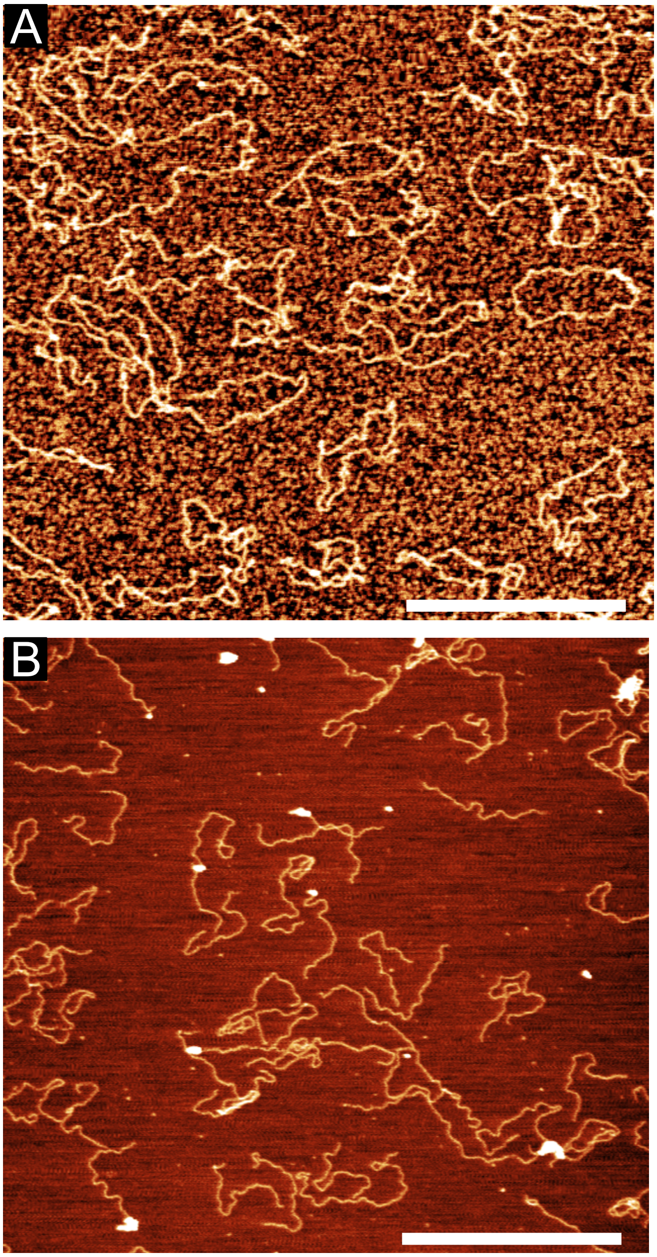

Figure S2. DNA imaged by tapping mode AFM. (A) 8000 bp supercoiled 601-197-25 plasmid. **(B)** 601-197-25 plasmid digested with EcoRV to yield 5000 and 3000 bp fragments. Scale bar 500 nm

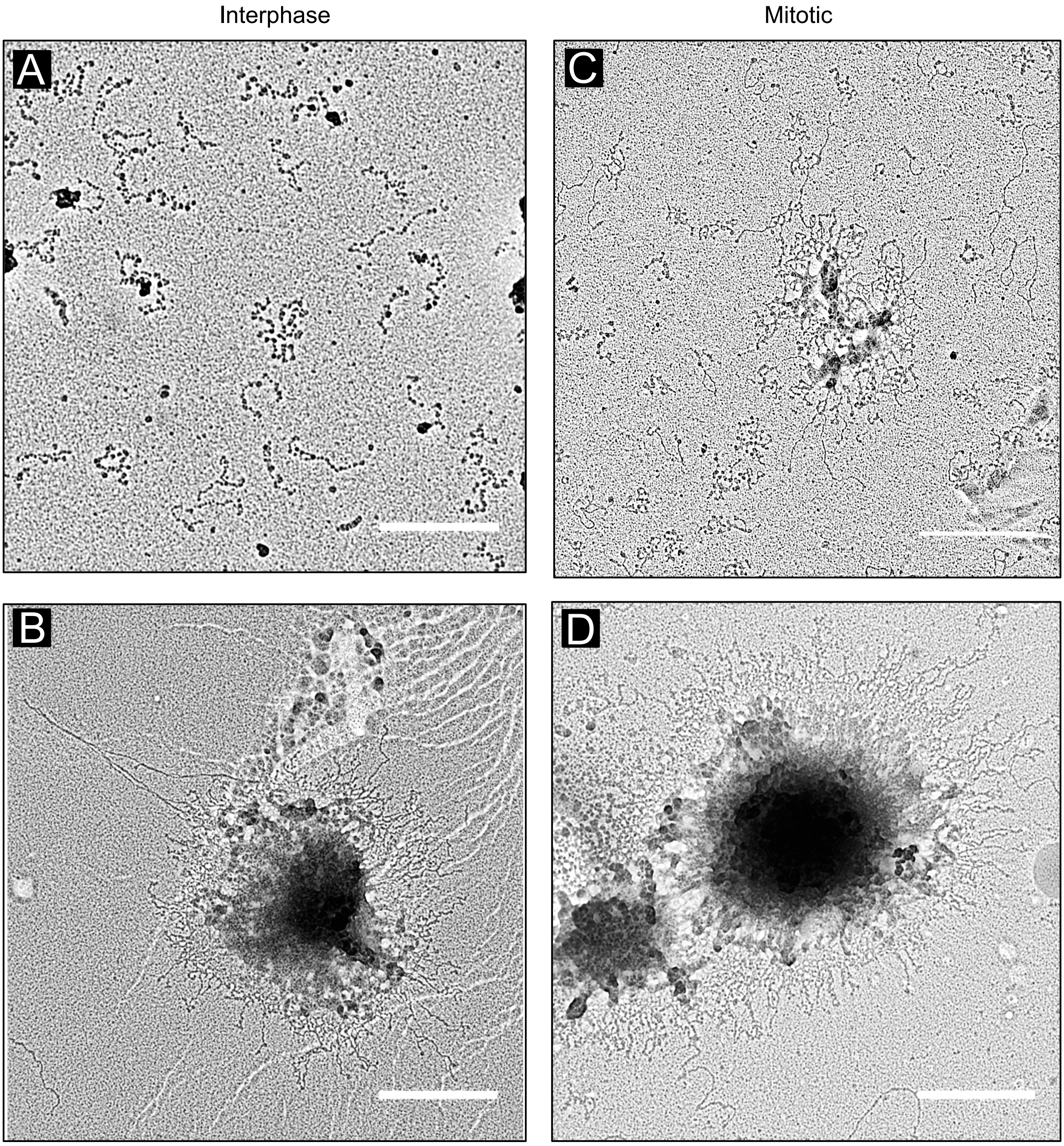

Figure S3. Transmission electron micrographs of reconstituted interphase and mitotic chromatin. (A, B) Chromatin reconstituted from interphase histones in the absence (A) and presence (B) of 10 mM Mg^{2+} ions. **(C, D)** Chromatin reconstituted from mitotic histones in the absence (C) and presence (D) of 10 mM Mg^{2+} ions. Scale bar= 500 nm
